# Learning indoor robot navigation using visual and sensorimotor map information

**DOI:** 10.3389/fnbot.2013.00015

**Published:** 2013-10-07

**Authors:** Wenjie Yan, Cornelius Weber, Stefan Wermter

**Affiliations:** Knowledge Technology Group, Department of Computer Science, University of HamburgHamburg, Germany

**Keywords:** spatial cognition, neural networks, robot navigation, cognitive system, environment learning

## Abstract

As a fundamental research topic, autonomous indoor robot navigation continues to be a challenge in unconstrained real-world indoor environments. Although many models for map-building and planning exist, it is difficult to integrate them due to the high amount of noise, dynamics, and complexity. Addressing this challenge, this paper describes a neural model for environment mapping and robot navigation based on learning spatial knowledge. Considering that a person typically moves within a room without colliding with objects, this model learns the spatial knowledge by observing the person's movement using a ceiling-mounted camera. A robot can plan and navigate to any given position in the room based on the acquired map, and adapt it based on having identified possible obstacles. In addition, salient visual features are learned and stored in the map during navigation. This anchoring of visual features in the map enables the robot to find and navigate to a target object by showing an image of it. We implement this model on a humanoid robot and tests are conducted in a home-like environment. Results of our experiments show that the learned sensorimotor map masters complex navigation tasks.

## 1. Introduction

Spatial cognition refers to humans' and animals' ability of gathering information about the environment, organizing and using the spatial knowledge, and revising it when the environment changes (Montello, 2001). It is a fundamental ability which helps us to achieve different tasks like navigation and grasping. The spatial knowledge of an environment is presented with features and relationships as a sensorimotor map, which enables an animal to navigate flexibly based on the abstract information stored in the sensorimotor map and to make detours by adjusting the map structure when an obstacle appears. Tolman (1948) observed rats not behaving in a simple stimulus-response fashion but based on some form of internal spatial representation of the environment, which he termed a “cognitive map”.

Considering that humans and animals can achieve various complex tasks, how information is processed in the brain should be the key for realizing an intelligent robot (Burgess et al., 2002). Therefore, the study of human spatial cognition is crucial for research on robot mobile behavior. A model based on spatial knowledge has the advantages of high robustness against sensor noise, good adaptation capability during environmental change, and high efficiency, which could overcome the challenges of indoor navigation such as high complexity of the environment and the possible dynamic changes during robot navigation. Furthermore, such a model supports the integration of a service robot into an ambient assistant living (AAL) setup.

Consistent with the suggestion that a spatial environment can be represented with sensorimotor features and actions associated with changes in the sensory input (Zetzsche et al., 2009), we develop a neural-inspired model for robot navigation based on learning sensorimotor representation of an indoor environment. The focus of this system is to bring into the real world a neural network for planning and navigation based on a model of spatial memory that resembles hippocampal place cells (Toussaint, 2006; Martinet et al., 2011).

The sensorimotor map consists of: (1) a spatial memory that learns the environment through visual perception, (2) an action memory for learning actions associated with state transitions in the spatial memory, and (3) an action layer that controls the robot's behavior based on the associated action input. While related models for navigation have only been tested in simulation (Toussaint, 2006; Martinet et al., 2011), we have further developed our model into a real world scenario. To handle sensor and actuator noise present in a real environment, the positions of the robot and navigation target are represented by multiple hypotheses. Based on these distributed representations in the spatial memory, multiple action memories associated with state transitions combine in the competitive action layer, which yields a robust and smooth control signal for navigation. To handle the dynamics of a real environment, a reflex-like behavior avoids obstacles based on the robot's sensor signals and reduces the corresponding action memory weights. Thereby the robot remembers the obstacles in its spatial memory of the sensorimotor map and avoids them pro-actively in the future. Weight reduction and recovery, together with dynamic space representations, enable life-long model adaptation. A further unique feature of our approach is that by anchoring the appearance features of the environment with the states in the spatial memory, visual associations are linked to specific locations in the map, which is inspired by biological evidence [e.g., visual landmarks helps desert ants to return home (Collett et al., 1998)]. This visual anchoring allows the robot to achieve complex tasks such as fetching an object by showing an image of it. Together, these developments bring a neural network model for navigation into a dynamic real environment.

The reminder of this article is organized as follows: Section 2 briefly reviews the findings of spatial cognition and the related computational models developed in recent years, and section 3 presents the idea and the goal of our model. Section 4 provides insight into the model and the details of each component. Section 5 describes the mechanism of robot path planning, navigation control, reflex-like obstacle avoidance and the adaptation of the map connections based on the feedback of obstacle avoidance. Finally, we evaluate the test cases in section 6 and summarize in section 7.

## 2. Related work

Recent advances in neuroscience provide insight about the neural mechanisms of spatial cognition in humans and animals. An important finding are the place cells in the hippocampus of rats by O'Keefe and Dostrovsky (1971) and Andersen et al. (2006). Their activity rate is strongly related to the rat's location in the environment. Later on, head direction cells were found in the rat's brain [first in the postsubicular cortex of the hippocampus (Taube et al., 1990a,b), then in other related brain areas (Mizumori and Williams, 1993; Chen et al., 1994; Lavoie and Mizumori, 1994; Taube, 1995; Blair et al., 1998; Cho and Sharp, 2001)]. These cells fire selectively when the rat faces a specific orientation and provide a signal of the rat's heading direction during navigation (Pennartz et al., 2011). Also, anticipatory head direction signals are found in the anterior thalamus (Blair and Sharp, 1995; Taube and Muller, 1998). Together, these cells constitute a coordinate system that provides a representation of the location based on the animal's internal position sense in the environment.

A neural map can be represented in different ways, for example as a topological map (Cuperlier et al., 2007; Martinet et al., 2011), a continuous attractor network (Samsonovich and McNaughton, 1997; Milford et al., 2004; Samsonovich and Ascoli, 2005), etc. Toussaint (2006) developed a model using a self-growing mechanism (Fritzke, 1995) that forms a map with a dynamic size, which is flexible for exploring an unknown environment. The RATSLAM model developed by Milford and Wyeth (2010) provides a nature-inspired way for mapping, which represents the spatial information in its pose cells by combining the internal sensing and the external vision perception. However, the experience map in RATSLAM builds mainly line-like trajectories in space rather than mesh-like representations of space, due to a strict rule to connect cells. This constrains the generation of flexible navigation.

To acquire robust robot navigation, Weiller et al. (2010) proposed an unsupervised learning method to learn navigation behavior associated with state transitions in an unsupervised manner and control the robot during navigation by selecting the action with the highest value. Weber and Triesch (2008) and Witkowski (2007) present neural network models that learn associations between adjoining states and the action that links them. In addition, closed-loop control models for other behaviors, e.g., arm reaching, apply similar methods of planning (Herbort et al., 2010). A drawback of these models is that the representation of the state space is hardwired, which means that they only work in a well-defined environment. The action model in these models is discrete, and the robot is controlled by a winner neuron's action signal. Hence, the executed action might not be accurate in the continuous real world because of the discretisation error, or a fine-meshed action space would be required, which increases the learning effort strongly.

Given a population code for state estimation, there will be different actions suggested by the network for the robot to take. In order to allow their integration, the action layer is implemented as a neural field. A neural fields model has been seen as a simple but effective way to model motion perception (Giese, 1998), and has drawn more attention in the robotic area because of its distributed representation and the dynamic integration of information (Cuperlier et al., 2005; Toussaint, 2006; Torta et al., 2011). For example, Erlhagen and Bicho (2006) achieved a goal-directed robot navigation behavior with real-time obstacle avoidance with dynamic neural fields (DNFs). Because of the distributed information encoding, i.e., the neural population coding, the DNFs approach is able to generate stable signals by updating the activation patterns with noise canceling.

## 3. Motivation of our neural approach

Our neural architecture models the spatial context, the reward signal, the decision making and the action response as a whole. The spatial knowledge of the environment is modeled with an internal representation, i.e., the sensorimotor map, which allows the robot to select actions dynamically and to adapt its strategy when the environment changes. Not only the state transition but also the actions corresponding to them are represented in the map. In order to accelerate learning and to avoid possible danger caused by the robot's active exploration, the map is built by observing the movement of a person using our localization method with a ceiling-mounted camera (Yan et al., 2011). This design has been chosen in the context of an AAL setup that uses a small socially assistive robot as a communication interface to the person (The KSERA project). The ceiling camera presents a cheap and little intrusive solution to localize a person anywhere within a larger room, even when the small robot cannot directly see the person. At the same time, it supplies high-level visual input about the robot location in allocentric coordinates, consistent with hippocampal place coding, activating directly the neurons in the spatial memory. This bypasses the need of learning a visual system that localizes from the robot's camera image, as has been done by Wyss et al. (2006). The localization input, which comes via particle filters (Yan et al., 2011), is compatible with distributed neural coding (Deneve, 2005; Huang and Rao, 2009; Wilson and Finkel, 2009).

The map building itself resembles “latent learning,” where there is no task during the exploring of the room by a person. When the navigation task is active, the sensorimotor map together with the reward signals from the target position support a model-based reinforcement learning, which guides the robot for maneuvering to the target. Because the robot control is not calibrated, but learned, the model can be applied easily in different rooms without camera calibration. Parts of this work have been presented in Yan et al. (2012b).

Our model tackles the challenge of applying a neural system in realistic settings, and uses a humanoid Nao robot as experimental platform. Other than pure simulation models that simplify the environment or model the system dynamics exactly without considering noise, our robot needs to navigate by finding a movement direction among 360° in our home setup, which requires further refinement to match requirements of real application. For example, in order to obtain a continuous robot control, our model represents the individual action for each state transition to obtain a high control accuracy with minimal memory requirement and uses a population code to represent the state in a probabilistic manner. Using this distributed representation, multiple state transitions are active at the same time and the corresponding action signals are merged together via a ring-form neural field. This results in a smooth and continuous action control, where actions are generated that did not occur during map learning. Moreover, the sensorimotor map does not need to predefine its state space, because it adapts itself using latent learning while a person explores the unknown environment. As no goal is needed here, the path planning is not learned for a specific target, which permits a flexible navigation behavior toward an arbitrary possibly moving target.

According to Penner and Mizumori (2012), the dorsolateral striatum (generates automatic behavior when appropriate) and the dorsal striatum (coordinates the goal-directed behavior) are important for animals to generate flexible navigation behaviors. Our framework models the dorsolateral striatum with a reflex-like behavior for interaction with the obstacles and updates the connectivity of connections in the sensorimotor map, which resembles the dorsal striatum to coordinate the path planning and provides a pro-active obstacle avoidance property. Consistent with the advice of using a hybrid control architecture (Murphy, 2000), our system fuses the path-based and behavior-based navigation in a constructive manner.

In order to build up a memory of the environment context, features of the current camera view are extracted and associated with the current state of the robot in the spatial memory. This memory is essential to accomplish complex cognitive tasks, for example to ground visual appearances to a location (Bellotto et al., 2008). In our case, a camera integrated in the robot's head captures the appearance of the environment during navigation. The anchored memory of objects is used to let the robot locate an object in an environment by showing a picture of it after the robot has observed the environment properly through its explorative navigation.

## 4. Architecture

**Figure 1:**
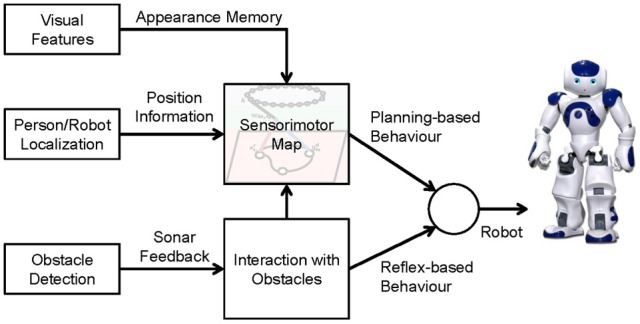
**Overall system architecture**.

The architecture of our navigation framework is shown in Figure 1. Three sensors are applied: (1) a robot camera fixed on the robot's head for extracting the visual features of the environment, (2) a ceiling-mounted camera to localize the person and the robot, and (3) sonar sensors installed on the robot's chest for detecting obstacles. The position information of the person and the robot is used for building up the sensorimotor map and planning navigation, and the visual features are used for generating an appearance memory that associates with the robot's position in the map. The interaction model generates reflex-based behavior for obstacle avoidance and also adapts the spatial memory so that the robot can remember and avoid the obstacle proactively next time.

**Figure 2:**
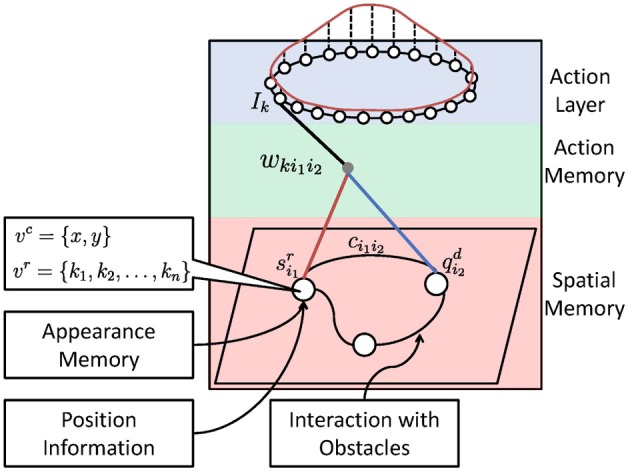
**Architecture of the sensorimotor map.** The red curve above shows the activation bump of the neural field (action layer) which is used for robot control.

The core of the system is the sensorimotor map. As shown in Figure 2, it consists of three components: (1) a spatial memory that learns the structure and the appearance of the environment, (2) an action memory that learns an inverse control model, and (3) an action layer for robot control. We will first describe these components and then the mechanism of planning and interaction with obstacles.

### 4.1. Spatial memory

The spatial memory layer represents the spatial information of an environment. It contains two types of spatial information: *states* that present the features and *connections* that present the relations between different features. When a person, as observed by the ceiling-mounted camera, or the robot visits a novel location, features of this location as well as the relation with neighboring places will be stored. The features of the spatial memory can be presented in different forms, for example, they could be readings of a laser scanner, etc. In our case, it is the position information (“where”) measured by the ceiling-mounted camera, combined with feature information (“what”) that is observed from the robot's camera. When the robot is at a specific position, the neurons whose features match with the ones obtained from the current position will be activated. This resembles the neural activity of the place cells in the hippocampus, whose firing rate is dependent to the current location.

During navigation, the desired next state will be estimated in the spatial memory layer to guide the robot to the target position (see Section 5). As output of the spatial memory layer, the activity signal from the current state of the robot, i.e., *q*^*c*^ (red line in Figure 2), and the desired next state, i.e., *q*^*n*^ (blue line in Figure 2), will be sent to the action memory layer. The computation of *q*^*c*^ and *q*^*n*^ is described in the following section.

The spatial memory is represented by a Growing When Required (GWR) network (Marsland et al., 2002). Compared with the Growing Neural Gas model (Fritzke, 1995) that we used in Yan et al. (2012b), GWR does not grow over time but only when novelty is detected, which provides better convergence properties. The network consists of a set *A* of neurons, each associated with feature vectors *v*, and a set *N* of connections to describe the relations (*i*_1_, *i*_2_) between neurons *i*_1_ and *i*_2_ in *A*.

The different features presented in the neurons are the *x*, *y* coordinate information on the image from the ceiling-mounted camera, *v*^*c*^ = {*x*, *y*}, and the appearance memory, which resembles the visuospatial perception, based on visual keypoints extracted from the robot's camera *v*^*r*^ = {*k*_1_, *k*_2_, …}. These features are used to determine the position of the robot as well as the target. Neurons and connections will be allocated or updated dynamically using a competitive Hebbian learning rule.

Because a person's walking behavior is different to the robot's, the map built by observing the person's movement might not be totally suitable for a robot. Hence, robot-environment interaction is essential for the robot to adapt its navigation strategy online. We therefore define a connection weight *c*_*i*_1_*i*_2__ ∈ [0, 1] for each connection (*i*_1_, *i*_2_) to indicate how “easy” a robot can move along this connection. The higher *c*_*i*_1_*i*_2__ is, the easier is the connection for the robot to walk through. When an obstacle is detected or the robot has difficulties walking further, *c*_*i*_1_*i*_2__ will be decreased and may reach zero. When a connection is created, its connection weight *c*_*i*_1_*i*_2__ will be initialized to 1 and adapted during the robot navigation. Details about this adaptation will be described in section 5. The following part of this section will explain the methods of map building based on the position and the visual features.

#### 4.1.1. Learning position information

The map building is based on the position information ξ = {*x*, *y*} from the person or the robot localization and orientation model using the ceiling-mounted camera [for details about the person- and robot-localization please see Yan et al. (2011, 2012a)]. We first find the winner neuron i^*^ and the second winner neuron i^**^ by calculating the map activity *s* (later we will use *s*^*p*^ to indicate a person and *s*^*r*^ to indicate a robot in section 5) based on ξ with the following equations:

(1) $$s_{i}=e^{-\frac{{\left\|v_{i}^{c}-\xi \right\|}^{2}}{2\sigma^{2}}}$$

(2) $$i^{*}=\arg\;\underset{{}_{i}}{\max}s_{i}$$

(3) $$i^{**}=\arg\;\underset{{}_{i\neq i^{*}}}{\max}s_{i}$$

A new node will be added between neurons i^*^ and i^**^ when the following two conditions hold:

1. The activity of the winner neuron *s*_i^*^_ is smaller than a threshold activity *a*_*t*_, which means that the person is far from the position represented by any map unit, and
2. The firing counter *h*_i^*^_ has become smaller than *h*_*t*_, which means this neuron cannot move a lot any more.

Neuron insertion includes the following steps:

- Insert a new neuron *r* with the average weights of the winner neuron and the current position:
  (4) A←A∪{r}
  (5) $$v_{r}^{c}=\frac{1}{2}\left(v_{i^{*}}^{c}+\xi \right)$$
- Insert edges between *r* and i^*^ as well as between *r* and i^**^
  (6) $$N=N\cup \left\{\left(r,i^{*}\right),\left(r,i^{**}\right)\right\}$$
- Remove the current connection between i^*^ and i^**^
  (7) $$N=N/\left\{\left(i^{*},i^{**}\right)\right\}$$

The coordinate information of the winner neuron (i.e., *v*^*c*^_i^*^_) as well as its neighborhood neurons (*v*^*c*^_*i*_ for all directly adjacent neurons of i^*^) will be updated. Each neuron is assigned an age factor *age*_*i*_, which can increase incrementally, and a firing counter *h* to control the adaptation efficiency. In order to improve the convergence of the network and to have a homogeneous distribution of the neurons, the adaptation is as follows:

(8) $$\Delta v_{{\text{i}}^{*}}^{c}=\epsilon_{i^{*}}h_{i^{*}}\left(\xi -v_{{\text{i}}^{*}}^{c}\right)$$

(9) $$\Delta v_{n}^{c}=\epsilon_{n}h_{n}\left(\xi -v_{n}^{c}\right).$$

where ϵ_i^*^_ and ϵ_*n*_ are the fixed learning rates of the winner and the neighborhood neurons, and *h*_i^*^_ and *h*_*n*_ are the corresponding firing counters. The firing counters (initialized with *h*_0_ > 0) are calculated as follows:

(10) $$\Delta h_{i^{*}}=\tau_{b}\left(\mu h_{0}-h_{i^{*}}\right)$$

(11) $$\Delta h_{n}=\tau_{n}\left(\mu h_{0}-h_{n}\right)$$

where τ_*b*_, τ_*n*_, and μ are constant parameters for controlling the adaptation. The firing counters indicate how “active” a neuron is. When a neuron is added in the network, its firing counter is initialized as a high value *h*_0_, which allows it to adapt its features quickly. During the iteration, *h* decreases toward a small value μ*h*_0_ and the neuron loses its mobility, which ensures that the neuron's positions become stable. After adaptation, we increase the age of all edges that connect with neuron i^*^:

(12) $${\text{age}}_{\left(i*,n\right)}={\text{age}}_{\left(i*,n\right)}+1,$$

and delete the connection whose age is over a threshold *age*_*m*_. Isolated neurons that have no neighborhood will be deleted as well. For details of parameter setting please see Table A1 in the Appendix.

#### 4.1.2. Anchoring the appearance memory

The robot's camera captures vision information when the robot walks during or after map building. The neuron i^*^ closest to the robot's location will be active in this case and the visual features extracted from the robot's camera will be assigned to *v*^*r*^_i^*^_:

(13) $$\begin{array}{l} k_{\text{new}}\{\cdot \}=\text{Extract}\_\text{Features}()\\ \;v_{i^{*}}^{r}\leftarrow k_{\text{new}} \end{array}$$

A buffer is defined for each neuron to store the last 64 visual features when the robot visits the corresponding place, irrespective of its orientation. We use SURF features (Bay et al., 2006) to present the information of keypoints (see Figure 6A). Each keypoint contains the *x*, *y* position of the feature point in the image of the robot camera and a 64-dimensional vector that represents the image gradients. As a result, the robot learns a memory by associating the extracted visual features with its current location in the spatial memory during navigation. When the robot visits the same place in the map again with different orientation, the features from the new point of view will be inserted to the same neuron corresponding to this place. This memory is used for locating an observed object by comparing the similarity between the features extracted from an image of the target and the visual features stored in the appearance memory. For details of computation of SURF features as well as feature matching please read the original paper (Bay et al., 2006).

### 4.2. Forward and inverse model

The forward and inverse model represents the robot control signals coupled with the state transition in the spatial memory layer. The action information is learned in the weights *w*_*ki*_1_*i*_2__ which connect fully with the neurons *k* in the action layer. Depending on the way of controlling the robot, the action information can be presented in a different form, for example the force, velocity, angle value, etc. In our case the robot is controlled by adjusting its heading direction. For this, we use a ring-form DNF, which will be described in the next section. During map building, when a robot moves in a room and its spatial representation changes from *i*_1_ to *i*_2_, the action executed at that time will be associated with this state transition. As a result, the robot learns the action for each state transition and is able to select the most appropriate behavior for navigation. We use the second order weights {*w*_*ki*_1_*i*_2__}, which is also called Sigma-Pi weights (Weber and Wermter, 2007), to store the action information associated with the state transfer in the spatial memory. When the robot shall move from *i*_1_ to *i*_2_ in the spatial memory, the corresponding second order weight will be activated with the input signal *s*^*r*^_*i*_1__ of the current state *i*_1_ and *q*^*d*^_*i*_2__ of the desired next state *i*_2_, and the output *I*_*k*_ toward unit *k* in the action layer will be computed as follows:

(14) $$I_{k}=\sum_{i_{1},i_{2}}w_{ki_{1}i_{2}}s_{i_{1}}^{r}q_{i_{2}}^{d}$$

where the calculation of *q*^*d*^_*i*_1__ will be described in section 5. Because of the distributed representation of the robot's position, multiple connections may be activated at the same time. Equation (14) sums up those inputs.

The neural field of the action layer has 36 nodes, hence *k* ∈ {1, 2, …, 36}. Assume that there are *m* neurons in the spatial memory layer, then the total number of connection weights *w*_*ki*_1_*i*_2__ are 36*m*^2^, which grows quadratically according to the number of spatial memory neurons. However, most of the weights are zero since the action layer is sparsely connected with the spatial memory layer.

These connection weights can be trained online. The action learning can be done based on observing the person's movement, or based on the robot's location and motion information. Here we describe the method of action learning only regarding the observation of the person's movement because in principle the action learning based on the robot's movement is similar. The winner neurons with respect to the person's position will be determined at first and all the connections *c*_*i*_1_i^*^_ between the winner neuron i^*^ and its neighborhood neurons (indexed with *i*_1_) will be adapted. Assuming that a connection *c*_*i*_1_i^*^_ is active, the direction associated with this connection is calculated and the corresponding weights *w*_*ki*_1_i^*^_ are trained. Since there are two possible walking directions for every connection (from *i*_1_ to *i*_2_ and from *i*_2_ to *i*_1_), we train both weights at the same time as follows:

1. According to the position (*x*_*i*_1__, *y*_*i*_1__) of neuron {*i*_1_} and (*x*_i^*^_, *y*_i^*^_) of neuron {i^*^} of the spatial memory, we calculate the possible orientation *o*_*i*_1_i^*^_ of connection *c*_*i*_1_i^*^_ using inverse trigonometric functions:
  (15) $$\begin{array}{l} \;\Delta x=x_{i^{*}}-x_{i_{1}}\\ \;\Delta y=y_{i^{*}}-y_{i_{1}}\\ o_{i_{1}i^{*}}=\arcsin\left(\frac{\Delta x}{\sqrt{\Delta x^{2}+\Delta y^{2}}}\right)\\ o_{i_{1}i^{*}}=\pi -o_{i_{1}i^{*}}\text{ if }\Delta y<0 \end{array}$$
  And then we calculate the opposite orientation *o*_i^*^i_1__:
  (16) $$o_{i^{*}i_{1}}=o_{i_{1}i^{*}}+\pi$$
2. Two bumps of activation with the size of the DNF will be created in the shape of a circular normal distribution, one around the link orientation:
  (17) $$p_{ki_{1}i^{*}}=\frac{e^{\kappa \cos\left(\frac{k\cdot 10\pi }{180}-o_{i_{1}i^{*}}\right)}}{2\pi J_{0}(\kappa )}$$
  and another around the opposite orientation:
  (18) $$p_{ki^{*}i_{1}}=\frac{e^{\kappa \cos\left(\frac{k\cdot 10\pi }{180}-o_{i^{*}i_{1}}\right)}}{2\pi J_{0}(\kappa )}$$
  where *p*_*ki*_1_i^*^_ is the *k*-th connection weight of the action memory for orientation *o*_*i*_1_i^*^_, κ is a constant and *J*_0_(κ) is the modified Bessel function of order 0 (Abramowitz and Stegun, 1965):
  (19) $$J_{0}(\kappa )=\frac{1}{\pi }\int_{0}^{\pi }e^{\kappa \cos(\theta )}\text{d}\theta$$
3. Minimize the errors between the current activation *p* and the second order weights *w*, i.e., $\frac{1}{2}||p_{ki_{1}i^{*}}-w_{ki_{1}i^{*}}|{|}^{2}$. Gradient descent leads to:
  (20) $$\begin{array}{l} \Delta w_{ki_{1}i^{*}}=\eta \left(p_{ki_{1}i^{*}}-w_{ki_{1}i^{*}}\right)\\ \Delta w_{ki^{*}i_{1}}=\eta \left(p_{ki^{*}i_{1}}-w_{ki^{*}i_{1}}\right) \end{array}$$
  where η is a fixed learning rate.

Note that the spatial map and the forward and inverse model is based on spatial relations, but not on temporal ones during person moving, because the person's motion commands are unknown. In case of learning the sensorimotor by observing the robot's movement, the forward and inverse model can be built based on the robot's action signal.

### 4.3. Action layer

The action layer generates the robot control signals based on the active action units during navigation. A DNF model is used to merge these action signals and to adjust the robot's walking orientation by showing the desired robot orientation.

The DNF is a biologically-inspired model of the neural dynamics in cortical tissues (Amari, 1977), which is of interest in robotics to generate dynamic behavior (Cuperlier et al., 2005; Erlhagen and Bicho, 2006). A one-dimensional ring-form DNF with 36 neurons is implemented in our work representing the desired robot orientation in 10° increments. The DNF is capable of integrating the multiple action codes received by the action layer and adjusting the robot's motion with a smooth orientation behavior. Each neuron *k* of the DNF has a membrane potential *u*_*k*_ that represents the activity and lateral connections *n*_*kj*_ with other neighbor neurons *j*. Through the following updating rule (Equation 21), the DNF generates an activation bump dynamically to show the suggested orientation for the next step.

(21) $$\tau \Delta u_{k}=-u_{k}+\sum_{j=1}^{36}n_{kj}f\left(u_{j}\right)+I_{k}+h$$

where *h* is a rest potential, τ is a temporal decay rate of the membrane potential, and *I*_*k*_ is the input stimulus of the *k*-th neuron received from the second order weights that encodes the desired robot orientation. We use here a Gaussian-function with negative offset as the function *n*_*kj*_ to describe the lateral interaction of neurons:

(22) $$n_{kj}=\beta e^{-\frac{{\left(k-j\right)}^{2}}{2\sigma^{2}}}-c$$

where β is a scaling factor, σ^2^ a variance, *k*, *j* the index positions of neurons and *c* a positive constant. The function *f*(*u*) is a sigmoid transfer function of a single neuron with a constant offset *g*:

(23) $$f(u_{j})=\frac{1}{1+e^{-\left(u_{j}-g\right)}}$$

The robot's desired orientation *O*^*d*^ is calculated using vector averaging:

(24) $$\hat{v}=\left(\begin{array}{l} {\hat{v}}_{x}\\ {\hat{v}}_{y} \end{array}\right)=\left(\begin{array}{l} \sum_{k}u_{k}\sin\left(\frac{10k}{180}\pi \right)\\ \sum_{k}u_{k}\cos\left(\frac{10k}{180}\pi \right) \end{array}\right)$$

(25) $$O^{d}=\{\begin{array}{ll} \arctan\left(\frac{{\hat{v}}_{y}}{{\hat{v}}_{x}}\right) & \text{if }{\hat{v}}_{x}>0\text{ and }{\hat{v}}_{y}>0\\ \arctan\left(\frac{{\hat{v}}_{y}}{{\hat{v}}_{x}}\right)+\pi & \text{if }{\hat{v}}_{x}<0\\ \arctan\left(\frac{{\hat{v}}_{y}}{{\hat{v}}_{x}}\right)+2\pi & \text{if }{\hat{v}}_{x}>0\text{ and }{\hat{v}}_{y}<0 \end{array}$$

We control the robot's navigation by giving it a differential orientation command:

(26) $$\Delta O=\{\begin{array}{ll} -c_{o} & \text{if }O^{d}-O^{p}>d\\ c_{o} & \text{if }O^{d}-O^{p}<-d\\ 0 & \text{else} \end{array}$$

where *O*^*p*^ is the present robot's estimated orientation (see Yan et al., 2012a), *c* is a constant rotation speed parameter and *d* is a constant threshold. Details about parameter setting are listed in Table A2 in the Appendix.

## 5. Planning and navigation

As described in Equation (14), the control signal entering the DNF network is computed based on the connection weights *w*, the activity of the current state *q*^*c*^ and the activity of the next desired state *q*^*d*^. Because the connection weights *w* are trained during map building and *q*^*c*^ can be computed with respect to the robot's position, this section focuses on how to determine the next desired state *q*^*d*^. We therefore assign for each neuron of the spatial memory a reward value that spreads from the target states representations iteratively with an exponential decrease. First, however, the navigation target needs to be marked in the spatial memory. Depending on the navigation task, we use the coordinate information *v*^*c*^ or the appearance memory feature *v*^*r*^ as initial signals to define the target. To this end, an initial reward *r*_*i*_(0) at the target location is calculated with the following steps:

1. Calculate the goodness-of-match signals *m*_*i*_ of the neuron *i* in the spatial memory. For approaching a person, we calculate the signals *m* based on the distance between the person's position ξ and the neuron's coordinate *v*^*c*^. Assume that the person's position is distributed with a position list ξ_*s*_ (indexed in *s*) with corresponding probabilities *w*_*s*_ where ∑_*s*_*w*_*s*_ = 1, the *m* is computed as:
  (27) $$m_{i}=\sum_{s}w_{s}e^{-\frac{{\left\|v_{i}^{c}-\xi_{s}\right\|}^{2}}{2\sigma^{2}}}$$
  This means, the closer *v*^*c*^_*i*_ of neuron *i* to ξ_*s*_ is, the higher the activity this neuron has. For finding an object, we compare the similarity between *v*^*r*^_*i*_ of neuron *i* and the features *v*_obj_ of the target object as follows:
  (28) $$m_{i}=\text{feature}\_\text{match}\left({\text{v}}_{\text{i}}^{\text{r}},{\text{v}}_{\text{obj}}\right)$$
  where *v*^*r*^_*i*_ are the learned keypoint features (see Equation 13) and *v*_obj_ are the keypoint features extracted from the target object. The more visual keypoints of the test object match *v*^*r*^_*i*_ of the neuron *i*, the higher the activity this neuron will have. If no matched feature is found, *m*_*i*_ = 0.
2. Normalize the match signals with a softmax function:
  (29) $${\tilde{m}}_{i}=\frac{e^{m_{i}}}{\sum_{i^{\prime }}e^{m_{i^{\prime }}}}$$
3. Assign *m*_*i*_ to initial reward signals with a threshold filter:
  (30) $$r_{i}^{p}(0)=\{\begin{array}{ll} {\tilde{m}}_{i}, & \text{if }{\tilde{m}}_{i}>{\text{threshold}}_{m},\\ 0, & \text{else } \end{array}$$
  where *p* indices the neuron of the initial reward signal.

Multiple units will contribute to localize the target object/person since the person's location is presented with a probabilistic distribution and the object features may be represented at different positions. For each *r*^*p*^_*i*_(0) > 0, the reward signals will spread separately to the neurons connecting to the neuron *i* (listed in *nl*) iteratively with a discount factor λ and the corresponding connection weight *c*_*ij*_ (see Equation 38):

(31) $$r_{j}^{p}(t+1)=\lambda c_{ij}r_{i}^{p}(t),\text{ for }j\in nl(t)\text{ and }r_{j}^{p}(t)<r_{i}^{p}(t)$$

where the neighborhood list *nl* will be updated for each iteration as follows:

(32) $$\begin{array}{l} \;n^{\prime }\leftarrow i\text{ if }i\text{ connects with neuron }j\in nl(t)\\ \text{ and }r_{i}^{p}(t+1)<r_{j}^{p}(t),i\notin nl(t)\\ nl\text{(}t\text{+1) = }n^{\prime } \end{array}$$

After the spreading phase, the final signal *r*_*j*_ of each neuron {*j*} will be calculated by summing up all the reward signals from the target's location distribution:

(33) $$r_{j}=\sum_{p}r_{j}^{p}$$

As shown in Figure 5, the gray-scaled brightness of the neurons indicates the reward spreading from the target location. The brighter the color is, the higher the reward this neuron has. Based on these reward signals, the robot plans its action by calculating the next position it should reach. Assume that the robot's position is represented by a group of neurons α in the spatial memory. The next possible position should be among the neighborhood neurons that connect with neurons in α directly. The activity *q*^*d*^_*i*_2__ of these neighborhood neurons *i*_2_, which connect with neurons *i*_1_ ∈ α, is computed as follows:

(34) $$q_{i_{2}}^{d}=\sum_{i_{1}\in \alpha }c_{i_{1}i_{2}}s_{i_{1}}^{r}r_{i_{2}}$$

where *s*^*r*^_*i*_1__ is neuron activity of the robot detection (see Equation 1) and *c*_*i*_1_*i*_2__ is the connection weight (see Equation 38). The higher *q*^*d*^_*i*_2__ is, the more desirable it is for the robot to be at this position. We scale *q*^*d*^_*i*_2__ to the range of [0, 1] by dividing all the *q*^*d*^_*i*_ with the maximal value max_*i*_(*q*^*d*^_*i*_). Theoretically, the distance of reward spread is unlimited, but the strength of the signal decreases exponentially with a constant discount factor, which leads to small gradients at large distances. For each decision making the robot only evaluates the states around the current location and uses a soft-max function to retrieve those with highest reward value. Neuronal noise would impose a limit at which the gradient toward the goal cannot be evaluated, but in the computer implementation, the limit will occur when the computer cannot distinguish the higher value due to the precision of the double float value. However, during our experiments this situation never appears.

### 5.1. Interaction with obstacles

An interaction mechanism is essential to provide the robot with some “reflex” behavior to protect the robot passively during navigation and to adapt the navigation strategy online. Since the spatial memory is built using a person's actual movement, it may well fit here, since it helps showing that some positions where the person can go are not accessible by the robot. The connection weights *c*_*i*_1_*i*_2__ in the spatial memory indicate how “easy” the robot can follow that link. They are further adapted depending on the interaction with the environment.

**Figure 3:**
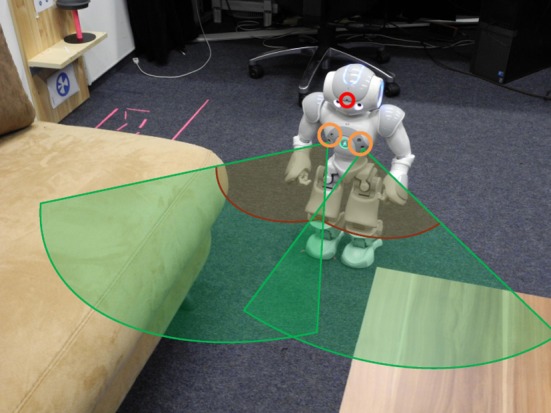
**Schema of robot sensors.** The robot uses a head camera (red circle) and two pairs of sonar sensors (orange circle on the chest). The detection ranges of the sonar sensors are illustrated via two green pies. Within the dark red line, the robot only knows that an object is present. Here, the robot cannot see the open space in front of him.

A small humanoid Nao robot is chosen for evaluating our architecture. It is equipped with various sensors, among them we use one camera in the head to observe the environment and the two pairs of sonar sensors for detecting obstacles during walking (Figure 3). Both sensors can detect the distance to obstacles robustly between 30 and 80 cm. A higher sensor value indicates a larger distance. Some routes learned from observing the person may be difficult for the robot to walk through, for example the path (shown in Figure 3) between the sofa and the tea table with a width of only 25 cm. Here, the sonar sensors indicate an obstacle in front of the robot. We simulate the sonar sensor in the simulator for modeling the obstacle avoidance behavior which is described in section 5.1.

In order to incorporate a reflex-like obstacle avoidance behavior, for each step, we compute a signal *G*(*s*_1_, *s*_2_) according to the sonar sensor signals *s*_1_ and *s*_2_ with a non-linear function:

(35) $$G(s_{1},s_{2})=\frac{a}{1+e^{-b(s_{1}+s_{2}-c)}}$$

where *a*, *b* and *c* are constant parameters. Two kinds of obstacle avoidance strategy will be triggered based on *G*(*s*_1_, *s*_2_). When *G*(*s*_1_, *s*_2_) is below a threshold, i.e., *G*(*s*_1_, *s*_2_) < γ, the robot will turn away from the obstacle based on the sonar signals:

(36) $$\Delta O=\{\begin{array}{ll} -c_{o} & \text{if }s_{1}>s_{2}\\ c_{o} & \text{else} \end{array}$$

where *c*_*o*_ is a defined parameter. When *G*(*s*_1_, *s*_2_) < 0.8 γ, the robot will walk slowly backwards besides turning.

The weight adaptation is as follows. When *G*(*s*_1_, *s*_2_) < γ, we update the connection *i*_1_*i*_2_^*^ with the highest value of *s*^*r*^_*i*_1__*q*^*d*^_*i*_2__, i.e., the connection along the current traveling direction:

(37) $$i_{1}i_{2}{}^{*}=\arg\;\underset{{}_{i_{1},i_{2}}}{\max}s_{i_{1}}^{r}q_{i_{2}}^{d}$$

Then, the connection weight *c*_*i*_1_*i*_2_^*^_ is adapted based on the sensor inputs:

(38) $$\Delta c_{i_{1}i_{2}{}^{*}}=\tau_{1}\left(G(s_{1},s_{2})-c_{i_{1}i_{2}{}^{*}}\right)$$

where τ_1_ is a learning rate. When the robot approaches an obstacle, the connection weight *c*_*i*_1_*i*_2_^*^_ will thereby be decreased and even converged to zero when the obstacle gets too close. Since obstacles may also be removed from the environment, we consider the following method for recovering the connection weights in this case. When *G*(*s*_1_, *s*_2_)> γ, all the connection weights around the current robot position are adapted with Equation (39):

(39) $$\Delta c_{i_{1}i_{2}}=\tau_{2}\left(G(s_{1},s_{2})-c_{i_{1}i_{2}}\right)\;\forall s_{i_{1}}^{r}>e$$

where *e* is a threshold of the distance and τ_2_ is a learning rate smaller than τ_1_. For our robot we adjust parameters as given in Table A3 in the Appendix.

## 6. Experimental results

**Figure 4:**
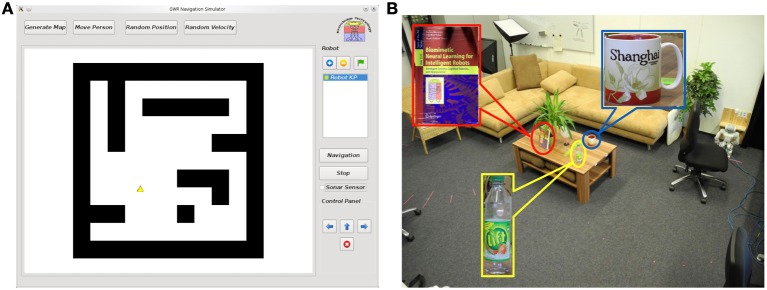
**Experimental setup. (A)** Simulator: The robot is displayed as a yellow triangle and the obstacles are shown as black blocks. Functions of map learning through moving the person's position (manually or automatically) and robot navigation toward the person's position are implemented. **(B)** Real test environment: A room containing some furniture with a size of 7m × 5m. Several objects are placed on the tea table for testing feature anchoring.

To evaluate the performance of our model, experiments are conducted in a simulator^1^ as well as in a real home-like laboratory. Figure 4A shows the GUI of the simulator. It mimics a real environment and is used for demonstrating the map building and the navigation functionality. We also simulate the mechanism of sonar sensors, which can be visualized. The setup of the real test environment is shown in Figure 4B. A ceiling-mounted camera with a resolution of 320 × 240 pixels is used to localize the person and the robot (for details please see Yan et al., 2011). A fish-eye lens gets a wide field of view of the whole room with a single camera but at the price of strong image distortion. Since the map building is based on the internal spatial representation from the ceiling camera view, this distortion does not interfere with the robot navigation behavior.

The following tasks are included in the experiments. First, the spatial memory is built by observing the movement of a person within the room. Second, once the map is built, the robot can either navigate to the target autonomously or be controlled by a joystick in order to learn the environment further. While the robot walks, appearance features are extracted from the robot's head camera (with a resolution of 320 × 240 pixels) and stored to the corresponding spatial state. Based on this memory, the robot is capable of locating and navigating to the target object when showing an image of it. Third, since obstacle avoidance leads to a decay of the spatial memory connection weights, we evaluate the performance of interaction with environments by comparing the navigation behavior before and after obstacle avoidance. Details are presented in the following sections.

### 6.1. Map learning by observing person movement

**Figure 5:**
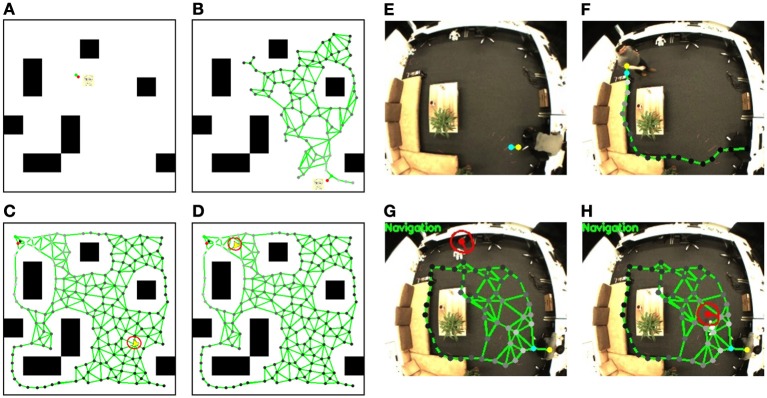
**Sensorimotor map learning and robot navigation in the simulator and the real test environment.** *Simulator*: **(A)** the initial status of the sensorimotor map, the person's position is simulated with mouse input. Black blocks are the obstacles that the person and the robot cannot walk through; **(B)** a sensorimotor map is growing based on the person's position; **(C)** a completed sensorimotor map of the traversable area is built and the robot starts navigating to the target position labeled with a red spot on the map; **(D)** the robot is approaching the target position. The robot in the simulator is displayed with a yellow triangle. The red circle shows the activity *u*_*k*_ of the DNF given by the action layer, and its bump visualizes the desired robot orientation. *Test environment*: The position of the target person is shown with a yellow (winner neuron) and a blue (second winner neuron) spot; **(E)** shows the initial map with two neurons and one connection; **(F)** shows the map growing based on the person's location; **(G)** shows the robot navigation toward the person based on the completed sensorimotor map. The red spot indicates the estimated robot position and the red short bar the estimated orientation; **(H)** the robot reaches the target person.

**Figure 6:**
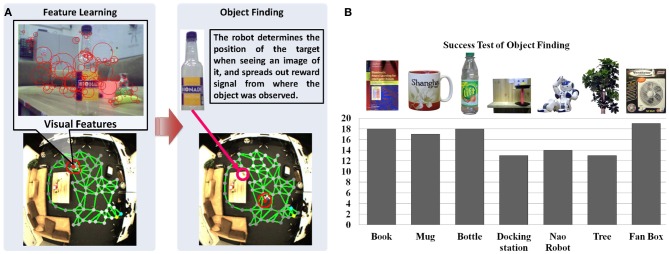
**Anchoring appearance features in the sensorimotor map. (A)** Shows the features extracted from the robot's camera during navigation. We use SURF features (Bay et al., 2006) for representing the appearance shown as red circles. **(B)** Shows the results of object finding. Each object is experimented for 20 times in our lab environment. The black columns show the number of successful tests and the success rate on average is 84.2%.

At the beginning of map learning, the spatial memory is initialized with two neurons linked with each other with a connection (see Figure 5E). Then, based on the person's position estimated by the visual input, the closest neuron to the person (winner neuron in yellow) and the second closest neuron (second winner neuron in blue) will be computed. The winner and its neighborhood neurons will be drawn to the person's position and new neurons will be inserted (see Figure 5F). The spatial memory will grow automatically when a person moves to a new place in the room, until most of the free space has been visited (Figure 5G). After the sensorimotor map building phase, we control the robot remotely to explore the room and to memorize the appearance of the environment. The visual features extracted from the robot's camera view (shown in Figure 6A) will be registered to the corresponding neuron where the robot is located. For details of the learning rules please see Equations (1–13).

### 6.2. Navigation to a person

The navigation task can be started after the map building. The robot will first be localized (see Figure 5G, the robot is localized with a red spot, and the red short bar shows its estimated orientation) and the neuron activities *s*^*r*^_*i*_ that represent the current position of the robot are computed. A reward signal then spreads from the person position using Equations (27–30) (see yellow spot) through the entire network with an exponential decrease, which is visualized through the brightness of the nodes (see Equations 31–33). The brighter the neurons are, the higher reward they have. Then, based on the current robot position and the reward signals in the sensorimotor map, the robot calculates the next desired state and generates the motion signals with Equation (14). The motion signals are merged in the DNF and the activities of the neurons of the DNF are updated (cf. Equations 21–23). In the lower row of Figure 5 we visualize the neuron activities of the DNF by a red circle surrounding the robot's position with a basic radius of 15 pixels where activations are zero, and a larger radius where activations are larger. An activation bump is built up which determines the desired orientation for navigation (cf. Equations 24–26). During the robot's movement, the state representations are changing and the activation bump will be updated to the new desired orientation. Also, when the target person moves, the reward spreading will be changed and the robot replans its behavior in real time^2^. Because of the dynamic behavior of the DNF, the robot will adjust its orientation slowly and walk in a natural way instead of reacting suddenly to noisy measurements. When the robot gets close to the person, the navigation will be achieved and the robot will stop walking (Figure 5H).

### 6.3. Object finding based on appearance feature memory

To test the object finding task, we show a picture of an object to the robot, which might have been observed by its head camera during the explorative navigation. Then, the robot searches its visual memory to find the states that contain the visual features resembling the features of the target object. As shown in Figure 6A, the reward signals are reset according to matching features extracted from the shown image and the features *v*^*r*^ of neurons (cf. Equation 13), and spread from the winner neuron to the entire network (see Equations 28–33). Then the robot can approach the object with the same method used for approaching a person^3^. Twenty experiments are conducted for locating different objects. For each experiment we first let the robot walk around the room and learn the appearance of the environment. Objects are placed in different positions each time, which are observed by the robot during navigation. After learning, we check if the robot can find the correct position of each object by showing an image of it. The object is considered to be identified correctly if the distance between the target node, i.e., the neuron with the highest reward in the spatial memory (see the brightness of neurons in the map) and the object is smaller than 30 pixels. As summarized in Figure 6B, the results vary with the objects. The docking station has the lowest success rate because of its simple structure and few detectable features. Due to the constraint of the robot hardware, the robot can provide images with 10 frames per second (fps) and features may be missing because of the image blur. The book and the bottle can be localized easily because sharp features can be extracted from their surfaces. Also, the light condition of the environment influences the experiments.

### 6.4. Map adaptation during navigation

**Figure 7:**
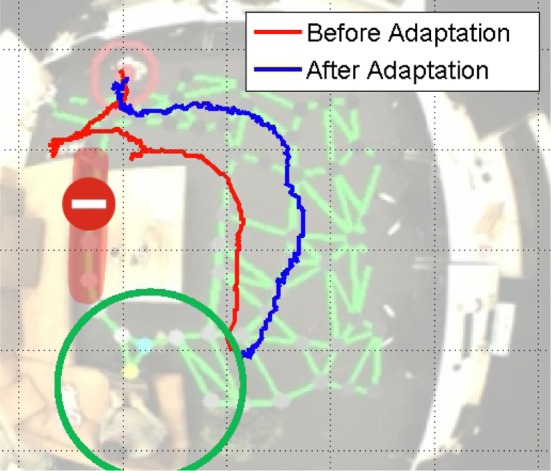
**Experiment of map adaptation.** The green circle denotes the target position. The area masked in red with a red ban sign shows the narrow path of 25 cm width, where the robot cannot walk through. The red and blue trajectories show how the robot navigates before and after the map adaptation.

The adaptation of the map during navigation is an essential ability of the robot to interact with the environment and adapt the spatial knowledge based on its interaction with obstacles. We set up a test scenario shown in Figure 7. The robot can navigate to the target position based on the map learned from observing the movement of the person, and among the connections in the map, the route masked in red seems better for navigation and the robot attempts to choose this way. However, since this narrow path has only 25 cm width, which is smaller than the limit of detection range of the sonar sensors, the robot is unable to walk through this path. The robot should then realize this difficulty by obstacle detection and adapt the sensorimotor map accordingly. We will start this task several times from the same position to check how the navigation performance improves.

The trajectories of different trials are shown in Figure 7. The robot first tries to navigate through the narrow path and fails due to the warning signal of the sonar sensors. According to this feedback, the corresponding connection weight decreases. At a certain point, when the connection weight is small enough, the robot's behavior will be changed. We then start the navigation from the same initial position again. As the trajectories of the first adaptation shows, the robot turns immediately to avoid the obstacle pro-actively. That means, the robot has learnt the suitable way for walking through map adaptation.

**Figure 8:**
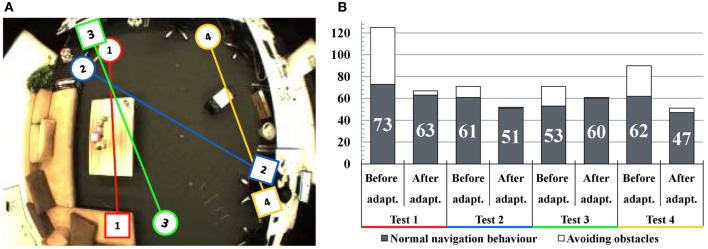
**Experiments of map adaptation through interaction with obstacles. (A)** Test scenarios of four navigation experiments. The circles denote the robot's starting positions and the squares denote the target positions. **(B)** Navigation time analysis. The vertical axis shows the consumed time for navigation in seconds. Black columns indicate the consumed time for normal navigation behavior and the white areas of the columns indicate the time spent while avoiding obstacles. In general, the time of obstacle avoidance decreases significantly after map adaptation.

We evaluate the performance of the navigation from different positions and a subset of the experiments is displayed in Figure 8A. A map is built each time and then we let the robot navigate to the person's position shown as squares in Figure 8A. Because the obstacles are detected by sonar sensors, the robot will keep a certain distance to the obstacles. Thus, the robot's behavior differs from a person's: the planned navigation paths are often blocked by corners of furniture which are easy for a person to avoid (see Test 1, 2, and 3). Also, a new obstacle was placed in the room (Test 4) which was not present during mapping. During the robot's own exploration, the robot detects the obstacles and adapts the map by deactivating the connections close to them. As a consequence, after the adaptation the robot would choose a safer route for approaching the target. In some cases (for example Test 3) the time for normal navigation behavior increases, because the robot may walk in a longer path to avoid obstacles. The time analysis of these experiments is shown in Figure 8B. As we can see, the total time of navigation decreases significantly and the run of the second adaptation is close to the manual control.

## 7. Discussion and conclusion

### 7.1. Summary

We have presented a novel neural framework for robot navigation in a cluttered environment based on sensorimotor map learning. The system consists of multiple layers of neural networks, which combine map building and localization with planning and navigation. The spatial memory is represented by a GWR network with self-organizing learning, which is related to dynamic [e.g., cell growth (Eriksson et al., 1998)] place cells in the hippocampus (Gorchetchnikov and Grossberg, 2007). The ceiling-mounted camera simulates a high-level visual perception model not only for robot localization and map building, but also person detection from an arbitrary position in the room. Our map learning and adaptation is inspired from the principles of sensorimotor learning (Wolpert et al., 2011): (1) observational learning that develops the map and the corresponding motor skills by watching a moving person and (2) error-based learning during navigation that adapts the map, and hence its navigation strategy based on interaction with obstacles. During navigation, the robot learns the appearance of the environment by anchoring the object's features to the corresponding neuron in the spatial memory, which simulates the visuospatial perception and enables the robot to combine the cognitive tasks of locating and navigating to an object held in memory. The navigation is planned in real time based on the reward signal spread through the spatial memory network from the target position. Since there is activity away from the robot's actual location, this could correspond to the activation patterns observed in hippocampal cells, which do not strictly encode the current position of a rat, but represent places it is considering to visit in the near future (Van Der Meer and Redish, 2010). The navigation tasks to different targets were achieved successfully in a home-like laboratory.

Our research represents the first adaptation of the kind of models of Toussaint (2006) and Martinet et al. (2011) to the real world, transferring the new concepts from a simplified to a real environment with higher complexity. Interesting further developments in our model have arisen from the needs of the real world. First, while the goal position and the current robot position can be determined perfectly in simulation, it becomes difficult in the real environment due to the uncertainty of the measurement. A distributed neural coding is therefore used (i.e., multiple hypotheses) to represent the positions of the robot and the target. This brings the model closer to the spiking and redundant population coding in real neurons, and is useful to represent the locations from which the robot can see an object, as the object may be observed from different positions. Second, unlike in the simulator where a state transition with a certain action can be modeled deterministically, the effect of an action may be uncertain due to the presence of noise. Therefore, in our model we back-propagate the rewards on the state level, which is corrected through observation during navigation.

Our model is able to perform a flexible navigation to reach an arbitrary target without pre-training with a fixed goal position. The robot moves within a broad home-like environment, which requires selection of a continuous direction among 360°, rather than discrete choices as in mazes or corridors. Hence, we merge different actions weighted with their corresponding activities to generate actions that are more precise and robust with respect to the discretization of the grid of the spatial map. Another aspect that needed to be considered for real-world integration is the focus on the obstacle avoidance and the adaptive map and planning behavior in a dynamic environment, which is an attractive topic in robotics. This allows perpetual learning which is required in a dynamic environment.

Overall, the key achievement of this work is the successful development of a neural model for robot indoor navigation and visual appearance anchoring to realize cognitive tasks such as finding and approaching an object. The obstacle avoidance validates the model in a dynamic environment, which requires to incorporate a simple reflex to unknown obstacles. To remember them in spatial memory leads to improved performance.

### 7.2. Outlook

The presented architecture learns a sensorimotor map through observing the movement of a person in a room in order to accelerate the mapping phase. However, it might not be suitable for a person to explore some places (for example a room with high temperature) for safety reasons. Accelerating the room mapping without support from a person is essential in this case. In future work we plan to therefore focus on learning a sensorimotor map via observing the active exploration movement of a robot. The ceiling-mounted camera is an effective external sensor that needs to be installed, but also constrains the flexibility of the system. We will therefore also consider to build up the map using only the head camera of the robot.

### Conflict of interest statement

The authors declare that the research was conducted in the absence of any commercial or financial relationships that could be construed as a potential conflict of interest.

## Footnotes

^1^Source code of the simulator please find at the following link: http://www.informatik.uni-hamburg.de/WTM/material/GWR_Navigation.zip

^2^For a demonstration of a robot following a moving person please see the video at: http://www.informatik.uni-hamburg.de/WTM/material/videos/navigation_following_person.mpg

^3^For a demonstration of visual feature learning, object fetching and carrying the object back to the person please see the video at: http://www.informatik.uni-hamburg.de/WTM/material/videos/demo_object_fetching.mpg

## Appendix

### Parameter setup

**Table A1:** **Parameter table of the sensorimotor map building**.

σ	20	ϵ_*i*^*^_	0.05
ϵ_*n*_	0.02	τ_*b*_	0.165
τ_*n*_	0.066	μ	0.09
*h*_0_	1	*age*_*m*_	50
*a*_*t*_	0.7	*h*_*t*_	0.5

**Table A2:** **Parameter table of the forward and inverse model**.

κ	0	η	0.2
σ	2	*g*	3
*d*	0.3	*c*	0.3
*c*_*o*_	0.3		

**Table A3:** **Parameter table of the obstacle interaction model**.

*a*	1	*b*	10
*c*	0.5	γ	0.45
*c*_*o*_	0.25	*e*	0.3
τ_1_	0.3	τ_2_	0.05
