# Supplementary figures and images for: Learning indoor robot navigation using visual and sensorimotor map information
Source: Front Neurorobot. 2013 Oct 7;7:15. doi: 10.3389/fnbot.2013.00015 (PMC3791472; doi:10.3389/fnbot.2013.00015)

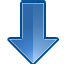

Supplement: Supplementary file 3 [file DataSheet1.ZIP › GWR_Navigation/Figs/down.png]

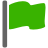

Supplement: Supplementary file 3 [file DataSheet1.ZIP › GWR_Navigation/Figs/Flag.png]

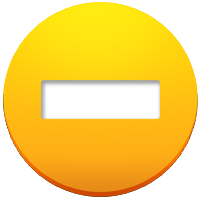

Supplement: Supplementary file 3 [file DataSheet1.ZIP › GWR_Navigation/Figs/remove.png]

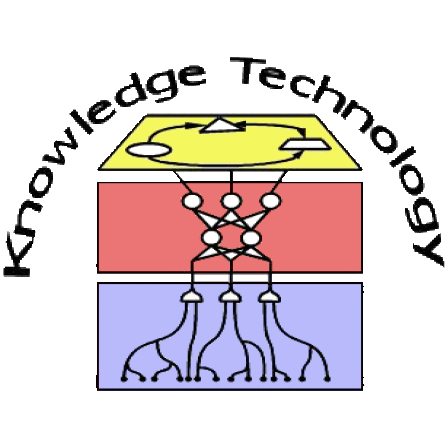

Supplement: Supplementary file 3 [file DataSheet1.ZIP › GWR_Navigation/Figs/wtmIcon.png]

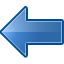

Supplement: Supplementary file 3 [file DataSheet1.ZIP › GWR_Navigation/Figs/left.png]

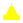

Supplement: Supplementary file 3 [file DataSheet1.ZIP › GWR_Navigation/Figs/KP.png]

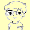

Supplement: Supplementary file 3 [file DataSheet1.ZIP › GWR_Navigation/Figs/person.png]

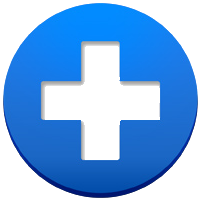

Supplement: Supplementary file 3 [file DataSheet1.ZIP › GWR_Navigation/Figs/add.png]

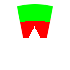

Supplement: Supplementary file 3 [file DataSheet1.ZIP › GWR_Navigation/Figs/sonar1.png]

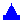

Supplement: Supplementary file 3 [file DataSheet1.ZIP › GWR_Navigation/Figs/KC.png]

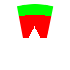

Supplement: Supplementary file 3 [file DataSheet1.ZIP › GWR_Navigation/Figs/sonar.png]

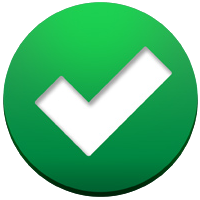

Supplement: Supplementary file 3 [file DataSheet1.ZIP › GWR_Navigation/Figs/yes.png]

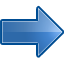

Supplement: Supplementary file 3 [file DataSheet1.ZIP › GWR_Navigation/Figs/right.png]

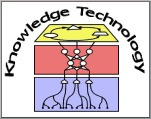

Supplement: Supplementary file 3 [file DataSheet1.ZIP › GWR_Navigation/Figs/KTiconS.jpg]

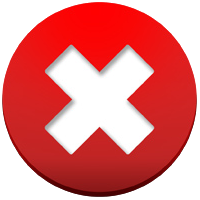

Supplement: Supplementary file 3 [file DataSheet1.ZIP › GWR_Navigation/Figs/no.png]

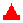

Supplement: Supplementary file 3 [file DataSheet1.ZIP › GWR_Navigation/Figs/KT.png]

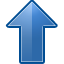

Supplement: Supplementary file 3 [file DataSheet1.ZIP › GWR_Navigation/Figs/up.png]
